# Evolutionary ethnobiology and cultural evolution: opportunities for research and dialog

**DOI:** 10.1186/s13002-017-0199-y

**Published:** 2018-01-09

**Authors:** Flávia Rosa Santoro, André Luiz Borba Nascimento, Gustavo Taboada Soldati, Washington Soares Ferreira Júnior, Ulysses Paulino Albuquerque

**Affiliations:** 1 Laboratory of Ecology and Evolution of Social-ecological Systems, Departamento de Botânica, Universidade Federal de Pernambuco, Cidade Universitária, Recife, Pernambuco Brazil; 2 Postgraduate Program in Ethnobiology and Conservation, Universidade Federal Rural de Pernambuco, Dois Irmãos, Recife, Pernambuco Brazil; 3 Departamento de Botanica, Universidade Federal de Juiz de Fora, Rua José Lourenço Kelmer, s/n, São Pedro, Juiz de Fora, Minas Gerais Brazil; 4 Universidade de Pernambuco, Campus Petrolina, BR 203 km 2 S/N, Vila Eduardo, Petrolina, Pernambuco Brazil

**Keywords:** Cultural transmission, Human behavior, Social learning, Social-ecological systems, Traditional ecological knowledge

## Abstract

The interest in theoretical frameworks that improve our understanding of social-ecological systems is growing within the field of ethnobiology. Several evolutionary questions may underlie the relationships between people and the natural resources that are investigated in this field. A new branch of research, known as evolutionary ethnobiology (EE), focuses on these questions and has recently been formally conceptualized. The field of cultural evolution (CE) has significantly contributed to the development of this new field, and it has introduced the Darwinian concepts of variation, competition, and heredity to studies that focus on the dynamics of local knowledge. In this article, we introduce CE as an important theoretical framework for evolutionary ethnobiological research. We present the basic concepts and assumptions of CE, along with the adjustments that are necessary for its application in EE. We discuss different ethnobiological studies in the context of this new framework and the new opportunities for research that exist in this area. We also propose a dialog that includes our findings in the context of cultural evolution.

## Background

Evolutionary ethnobiology (EE) examines the relationship between people and biological resources by investigating cognitive and behavioral characteristics within ecological and evolutionary frameworks (see [1, 2]). Specifically, EE involves studies of social-ecological systems, which briefly, can be understood as the dynamic interactions between culture and the environment (see [3] for more about social-ecological systems). These interactions result in a body of knowledge, actions, and beliefs within human populations that can be socially transmitted and subjected to selective pressures over time; this process is called traditional ecological knowledge [4].

Although some researchers have focused on evolutionary, structural and functional studies of social-ecological systems in ethnobiology [5–7], the field of evolutionary ethnobiology has only recently been formally conceptualized [8], and it addresses specific objects of interest [1]. Albuquerque et al. [1] argue that several disciplines that focus on the evolutionary aspects of human behavior may support theoretical developments and guide advancements in this field; these approaches include environmental psychology, evolutionary ecology, ecological anthropology, human behavioral ecology, evolutionary psychology, and cultural evolution. The field of cultural evolution (CE) regards culture as a fundamental cause of non-genetic behavioral variations among people and suggests that these variations can be viewed from a perspective that is similar to that of biological evolution (see [9]).

The evolutionary perspective is essential to the growth of ethnobiology as a science. According to Albuquerque et al. [1], ethnobiology still focuses on lists of useful natural resources, which is of great importance because it records knowledge that may be lost by local populations; additionally, this knowledge can be useful in the search for new remedies and other useful products, but this approach fails to identify patterns in the use of such resources and may be insufficient for developing the theoretical foundation of ethnobiology. Considering the forces that helped shape the complex relationship between humanity and natural resources will help us move forward in building theories in ethnobiology.

This article introduces cultural evolution (CE) to investigators who are interested in EE. Furthermore, it specifies possible research opportunities and presents potential topics for dialog that include EE and CE. The text is organized to describe the most basic concepts of cultural evolution and to contextualize them by considering the interests of EE whenever possible.

## CE: basic assumptions

We know that there are several different theories of “cultural evolution” and that this term can be understood in the context of controversial concepts, from progressive theories in the social sciences to new biological theories, such as memetics (see [10] for memetics). Therefore, we have to specify that when we refer to “cultural evolution” (CE) here, we are referring to a discipline that initially started with studies that used mathematical models to explain human behavior, by authors such as Luigi L. Cavalli-Sforza and Marcus W. Feldman [11] and Robert Boyd and Peter J. Richerson [12]; most recently, the study of cultural evolution has been widely discussed by those authors and others such as Joseph Henrich, James Broesch, Gillian R. Brown, Kevin Laland and Alex Mesoudi (see [9]). Therefore, here, cultural evolution is not only one process; rather, it is a discipline that addresses various complex cultural evolutionary processes.

CE assumes that, in addition to genetic information (genes), humans are entities who can store, handle, and express another form of information, i.e., culture [9]. In this sense, culture may be understood as information that is acquired through social transmission, such as teaching and imitation (rather than by genetic transmission) [9, 11–14]. Thus, in CE, information is employed as a broad term that incorporates ideas, knowledge, beliefs, values, skills, and attitudes [15].

From the cultural evolution perspective, culture can be viewed as a form of human adaptation [13, 14, 16, 17]. Human behavior is, therefore, the result of the expression of both genetic and cultural information. Although behavior results from the expression of information, not all information will necessarily determine a behavior because the information may not be expressed.

Although CE considers the co-evolutionary relationships between genetic and cultural information systems, it focuses on the dynamics of cultural traits. Cultural traits are the units of transmission of cultural information that can manifest in behaviors and other forms of communication, such as language [18]. A cultural trait can be measured when we observe a transmission event. For example, the words of an idiom are cultural traits that can be measured when two people use the local vocabulary. The ability to learn cultural traits is a primary factor that is responsible for the evolution of behavior [19].

The main assumption of CE is that the structural dynamics of cultural systems can be described using the basic Darwinian assumptions of *variation*, *selection*, and *heredity* [9, 20]. For cultural evolution to occur, it is necessary for cultural traits to vary, either at the individual level (i.e., one person holds two or more competing traits) or at the group level (i.e., different traits between and within human groups). Genetic variations within populations are essential to evolutionary processes, especially in response to environmental disturbances. The same occurs in cultural evolution. However, the sources of cultural trait variations within a given population are not always random; this is unlike the genetic mutations that proceed according to neo-Darwinian evolution, which can produce new phenotypes. In cultural evolution, individuals can voluntarily modify the information that they receive [9, 21].

This variability leads to the preservation of certain cultural traits over others due to *competition* for expression and attention because some traits are more likely to spread within a cultural or environmental context than others. This type of competition is not a form of direct competition, such as that between animals that are actively competing for food; rather, it is an indirect form of competition, with several traits that have greater probabilities of being learned than others (i.e., *differential fitness*) (see [22, 23]). This competition is stronger and more evident between similar types of information [9]. For example, in experiments of read and recalled words, people had more difficulty remembering different words that have the same meaning than those with different meanings [24]. This may occur because coexistence within the same cultural domain (see [25, 26] to cultural domain) is highly competitive. In ethnobiological studies, we can treat natural resources used for the same purpose (such as those used for firewood, or those used for the same disease or similar diseases) as those belonging to the same cultural domain.

Finally, for traits to become fixed within a population, they must be *heritable* (i.e., transmittable to other individuals). Like most genetic traits (excluding several bacterial traits), cultural heredity can be vertically transmitted from parent to child. Transmission can also occur through non-parental transmission pathways (i.e., between individuals of the same generation) [9, 12, 27, 28]. Together with the possible occurrence of several transmission events in a single individual (see [29]) or in a single cultural domain, these different transmission pathways allow for drastic cultural changes to occur within one generation [9, 13, 30]. The time it takes for a specific piece of information to become fixed within a population depends more on the cultural transmission pathway between each person than on the inter-generational time intervals. In this way, the cultural trait system is more flexible and dynamic than the biological system of genetic information. Although these characteristics promote the quick spread of adaptive responses to rapidly changing environments, they can also make the system more susceptible to the spread of maladaptive traits (see [31]), which are addressed below.

A key component of cultural evolution is the transmission of cultural traits through the expression of information (i.e., the transmission of a behavior in a manner that is analogous to a phenotype) [9], which makes cultural transmission much more complex than genetic transmission, where the genotype is transmitted. Because people can modify what they learn (phenotype) from others before they pass that information on, inheritance in cultural evolution can be described as *Lamarckian* [9]. Therefore, although they are very similar, there are differences between human genetic evolution (particularly from the widely studied neo-Darwinian perspective) and cultural evolution (Table 1).

**Table 1:** General characteristics of genetic and cultural evolution (adapted from [9, 12])

Evolution type/characteristics	Human genetic evolution (neo-Darwinian)	Human cultural evolution (Darwinian)
Evolutionary time	Usually thousands of years	Short time intervals, may occur within a few years
Variation	Random but some regions of DNA are more susceptible to mutation than others	Can be random or voluntarily guided by learning rules
Competition	Differential fitness of different alleles	Differential fitness of different cultural traits
Heredity	Parental Information storage structure (genotype) is transmitted	Parental or non-parental Information expression (equivalent to a phenotype) is transmitted

The principles of variation, competition, and heredity are primarily and widely studied in analyses that aim to understand the microevolution of culture. Cultural microevolution attempts to understand the factors and processes that promote changes in the frequency of cultural traits within a human population [9]. This approach mainly uses mathematical models [11, 12, 32], laboratory-based studies (i.e., psychological experiments) [33, 34], and field studies (similar to studies in ethnobiology) [35, 36] to predict the behaviors of different cultural traits and their selection over time.

These population-level microevolutionary patterns can be used to reconstruct the large-scale, long-term patterns and trends of cultural evolution from a macroevolutionary perspective. The macroevolutionary approach of CE resorts to comparative methods or phylogenetic methods [9] to understand the convergent and divergent evolution of traits in different populations worldwide, as well as the temporal origins of several important cultural traits of modern societies. A study by Salis-Lagoudakis et al. [37] is one example that uses this approach. This study shows that unrelated and geographically distant human populations (on different continents) select phylogenetically similar medicinal plant species for similar therapeutic ends. These results indicate convergent evolution, as these populations do not appear to descend from a common ancestral society.

Important ecological and evolutionary questions may underlie the relationships between people and their natural resources, which are investigated in ethnobiology. However, with few exceptions (see [6, 30, 38]), ethnobiological studies do not typically consider the evolutionary perspective. Nevertheless, several patterns that are observed in ethnobiology can be understood and studied from a CE perspective, particularly those that are associated with microevolutionary processes. These processes are the focus of this article because they allow us to draw several parallels with EE studies and to present new perspectives. The key concepts of CE and how they can be applied to EE are shown in Table 2.

**Table 2:** Key concepts of CE and their application to EE (adapted from [1, 9, 15])

CE characteristics	Definitions	Application to EE
Culture	Socially transmitted information that can affect individual behaviors.	The focus is not on culture as a whole but on the information that is associated with social-ecological systems and is expressed in the relationships between people and biota [8].
Cultural traits	Cultural information that can be discretely or continuously transmitted.	EE can investigate and quantify cultural traits to generate hypotheses. An example of quantifiable cultural traits is therapeutic targets and the medicinal plants used to treat them (see [7]).
Variation	Heterogeneity of cultural traits within the group and between individuals.	EE can study the real and potential heterogeneity of cultural traits within a cultural domain (i.e., the redundancy (variety) of medicinal plants to treat a disease) [40].
Innovation	Introduction of a new cultural trait that results from different processes, such as the individual production of knowledge, guided variation, migration, or erroneous social transmission.	Innovation increases the heterogeneity of social-ecological systems, which is the basis for cultural evolution. For example, exotic species may be introduced into the social-ecological system by immigrants [48].
Individual production of knowledge	A type of innovation; a process by which an individual builds new information (innovations), particularly through experimentation; this new information may or may not be transmitted or become fixed within the culture.	EE can investigate if a cultural variation originates from the individual production of knowledge or another source of innovation. For example, local medical specialists can create new remedies by aggregating cultural traits within a local medical system (i.e., the cultural domain) [6].
Differential fitness	Characteristics that increase the appeal of learning a given cultural trait.	Some traits are more appealing or transmittable than others. Additionally, traits that confer adaptive advantages in social-ecological systems can be prioritized to be copied. For example, in a local medical system, information on the treatment of frequent diseases is more memorable than information on others [41].
Lamarckian inheritance	Modifications to the expression of a cultural trait (equivalent to a phenotype) are transmitted during social transmission.	This characteristic allows for variations that are generated through guided variation in social-ecological systems to be transmitted to other individuals.

## Microevolutionary processes in ethnobiology

Mesoudi [9] subdivides the microevolutionary processes (Table 3) as follows: those that are related to variation and to the migration of a cultural trait; those that are related to cultural selection, which determines the probability that a trait will remain and be transmitted over other traits; cultural drift, such as the fixation of cultural traits through random processes; and those that are related to cultural transmission.

**Table 3:** Microevolutionary processes in cultural evolution (adapted from [9])

Processes	Description
Variation	
Cultural mutation	Randomly generated innovations, similar to genetic mutations
Guided variation	Individuals modify acquired information according to individual cognitive biases (Lamarckian)
Migration	
Demic diffusion	Cultural traits spread as their bearers move between different groups
Cultural diffusion	Cultural traits spread across group boundaries due to cultural transmission
Cultural selection	
Content bias	Preferentially adopting traits based on their intrinsic attractiveness (i.e., those that present strong emotional reactions)
Model-based bias	Preferentially adopting traits based on the characteristics of the model (person) (i.e., his/her prestige, age, or similarity)
Conformity bias	Preferentially adopting a trait based on its frequency (i.e., its popularity)
Cultural drift	
Random changes in cultural trait frequencies	
Transmission	
Pathway	
• Vertical	Transmission from the biological parents (uniparental or biparental)
• Oblique	Transmission from unrelated members of the parental generation
• Horizontal	Transmission from unrelated members of the same generation
Scope	
• One-to-one	Face-to-face learning from one individual to another
• One-to-many	One individual influences many individuals through mass education or mass media
• Many-to-one^a^	One individual is chosen to be taught by many experienced individuals
Mechanism	
• Blending	Adoption of the “average” of a continuous trait from more than one model
• Particulate	All-or-nothing transmission of discrete cultural traits

^a^Mesoudi [9] does not consider this transmission scope. Other researchers, such as Hewlett and Cavalli-Sforza [27], acknowledge the importance of the many-to-one scope

These microevolutionary mechanisms are only made possible by the human ability to learn and accumulate information. Social learning (through knowledge-transmission) decreases the amount of expended energy during the acquisition of new adaptive information and allows the diffusion of innovations [39]. However, an innovation caused by the individual production of knowledge can be more advantageous than a transmission in an environment with low stability because adaptive information at time *X* can cease to be adaptive at time *Y* [16, 19]. In social-ecological systems, the balance between cultural trait transmission and innovation, which is associated with environmental variations, determines the systems’ evolution through the selection and accumulation of adaptive cultural traits over time [19].

Questions that address microevolutionary processes that promote increases in information diffusion and variation can be thoroughly investigated in ethnobiology. These questions include the following: Why does the use of different natural resources utilized for the same purpose (redundancy) persist over time? Is local knowledge being lost over time? If so, is this decrease in local knowledge associated with a greater diffusion of other knowledge systems? In a given population, can socioeconomic factors promote the diffusion and/or inhibition of cultural traits that involve the use of natural resources? Few studies have used CE as a theoretical framework to understand these questions. The use of CE may allow significant advances in ethnobiology because such phenomena can be explained by the different processes that guide cultural changes.

### The importance of variation in microevolutionary processes

Utilitarian redundancy is a concept that emerged in ethnobiology that is used to characterize the organization and dynamics of local ecological knowledge [7, 40–45]. According to the utilitarian redundancy model, different biological resources may have the same utilitarian functions in a given social-ecological system. The redundancy of resources with the same function decreases the use pressure on the used species and increases the resilience of the social-ecological system (see [44]). For example, in local medical systems, some diseases may be treated by more than one resource, so these resources are redundant in terms of their therapeutic roles. Therefore, in the absence of one resource, another resource can be used in its place, which ensures the resiliency of the medical system and the maintenance of local health [40, 44]. This redundancy indicates a *variation* in traits within the same cultural domain, which is extremely important to the evolution of systems. We can draw a parallel to standing genetic variation, which shows that a species that maintains genetic variation at the same allele adapts faster [46, 47]. Therefore, standing variation has an important role in facilitating a swift adaptation to novel environments or rapid changes [47].

As previously mentioned, redundancies or trait variations in different cultural domains can voluntarily occur in cultural systems by guided variation. Guided variation, which Mesoudi [9] refers to as a Lamarckian microevolutionary process, occurs when information is intentionally modified by an individual to achieve a given objective. For example, someone learns about a palm tree species that is used to make a specific object, but then an environmental variation makes that species of palm tree unavailable at a particular time. This unavailability may cause the individual to use the original received information (i.e., the shape of the palm leaves) to experiment with using a similar species. In this way, the initial information a plant *X* can be used to make the object can be intentionally modified, which results in the addition of a new plant. Together with the original information, this new information may be transmitted and become part of a set of plants that is used by the community. This process of guided variation, when repeated several times, can increase the repertoire of useful plants for a given cultural domain. Therefore, as noted by Boyd and Richerson [12], guided variation does not depend on a previous variation in the population. Unlike the selection-like content and context biases, guided variation is related to an individual’s transformative trait and is unrelated to the cultural variants of other individuals.

However, the creation and recreation of a new trait can randomly occur through transmission errors or randomly generated innovations through a process known as cultural mutation [9]. These errors randomly occur, and the resulting damage or advantage of the modification is not perceived. When a given trait is transmitted multiple times, the probability that an error will occur increases; a higher frequency of knowledge transmission can generate noise in a similar manner to what occurs in the game of “Chinese Whispers.” This form of cultural mutation can also explain the results of ethnobiological studies of local medical systems that show a higher redundancy in medicinal plants that treat the most frequent diseases compared to those that treat infrequent diseases [7, 41]. These differences in treatment redundancy may occur because the higher frequency of the disease leads to a higher number of knowledge-transmission events regarding its treatment, which generates noise and becomes aggregated in the local pharmacopeia. In other words, the more times people have to transmit certain information, such as the treatment of a disease that occurs frequently, the more susceptible this information will be to random changes due to the same logic as that in the game of “Chinese Whispers.” However, the permanence of these errors is typically lower if they decrease the fitness of the population. If an error caused by cultural mutation decrease the fitness of the population, as in a case that leads to death, the local people will detect that the treatment information may not be useful and should be abandoned, even if the information is never perceived as the result of transmission errors. Thus, depending on the effect on the population’s fitness, this “erroneous” information can be eliminated, even if it is never perceived as the result of transmission errors. Relative to non-serious diseases, serious diseases often have fewer distinct medicinal plants that can be used for their treatments [7, 41], which may be due to the rapid elimination of variations in random treatment information when the threat to life is higher. For non-serious diseases, the errors may remain unidentified as errors because they have little impact on the population’s fitness. However, these explanations are mere assumptions because these ethnobiological studies do not focus on explaining the observed patterns within the cultural evolution framework. It is possible that these results simply show that fewer remedies are effective against serious diseases or that the frequency of the disease enables more experimental attempts with new medicinal plants, which increases the plant repertoire for frequent diseases. This observation highlights an adaptive characteristic in the local medical system. Thus, we must emphasize the importance of pursuing a more thorough analysis of these observations.

Migration is another source of cultural trait variations because it increases the trait variability within a culture by introducing new information [9, 13]. In CE, migration refers to both the migration of an individual who carries information from one place to another (i.e., demic diffusion) and the migration of the information itself (i.e., cultural diffusion) through other transmission pathways, such as books, radio, television, and the internet [9] (Table 3). Ethnobiological research contains numerous examples of cultural demic diffusion. These examples show that people carry with them the knowledge of their place of origin after migration and can exchange this information with people at the new site, adopt novel knowledge, and create variation [48–51]. However, cultural differences can block the exchange of information, thereby preventing the increase in cultural variation [52, 53].

One example of a migration-associated variation is the introduction of an exotic species into a local pharmacopeia. By demic diffusion, a person migrates and carries a plant with him; by cultural diffusion, the person sees information about the plant on television or in a magazine and acquires it at a local market. Albuquerque [54] suggests that this introduction may involve the need to fill the treatment gaps that are left open by the native species. Therefore, a possible interpretation in CE is that migration creates variation, which fills the local cultural domains that were previously left open, or introduces variants that increase the local population’s fitness, which favors the fixation and spread of these new traits over time.

Another example of cultural migration that is widely studied in ethnobiology is the introduction of western medical systems, (i.e., healthcare centers where industrial drugs are available) into a local medical system that is based on medicinal plants and has different definitions of illnesses. Several studies have suggested that the presence of western medicine (information that migrates into the community) has a negative impact on preexisting medicinal information [55, 56]. However, these studies do not regard the evolutionary perspective, which might contribute to the interpretation of data. For example, if these drugs present an advantage to the population (i.e., by increasing its cultural or biological fitness) and if they are compatible with the preexisting system, they can coexist with the local medical system (see medical pluralism in [57, 58]). Other ethnobiological studies have introduced various new issues related to migration, though they have not originally approached the evolutionary issue (Table 4).

**Table 4:** Selected factors that affect knowledge distribution and/or transmission in social-ecological systems and the possible contributions of cultural evolution (CE) to their study

Processes and factors that affect natural resource knowledge and use	Central questions in ethnobiology	Examples of studies with this focus	Contributions of CE to the understanding of these questions	Questions for future studies
Population migration	How does the knowledge repertoire of migrant populations vary in newly occupied environments?	[48, 52, 92, 93]	Human migration promotes the migration of cultural traits and increases the variation in information; it can also homogenize a population by eliminating different equilibria.	Which transmission biases affect migrant populations when they move to a new environment? How does the transmission between native and migrant populations occur? Is the rate of guided variation higher in a migrant population?
Gender	Does traditional ecological knowledge vary between genders?	[72, 75, 77]	Model biases may lead individuals to copy only people of the same or opposite gender.	Are there cultural traits that are preferentially copied from women? From men? “Does knowledge homogeneity vary between genders?”
Age	Do older people possess more local ecological knowledge? Does this phenomenon result in a loss of information in the population over time?	[71, 72]	Model biases may lead individuals to copy older people because their age and life experience provide a stronger knowledge framework. However, this greater knowledge does not necessarily indicate an information base that confers the highest fitness. Younger people may possess less information, but this phenomenon may represent gradual changes in the information repertoire rather than less knowledge.	Are there cultural traits that are preferentially copied from older people? Does the knowledge differ between younger and older people? Do the cultural traits that are exhibited by older people have a lower adaptive fitness in the current environment?
Ethnicity	Do different ethnic groups develop different ways of relating with the available resources?	[92, 93]	Several cultural selection biases, such as model biases, lead to heterogeneity of information among different ethnic groups that share the same environment, while other cultural biases that relate to environmental responses, such as content biases, lead to a homogenization of cultural traits among different ethnic groups that share the same environment.	Are the differences among different ethnic groups that share the same environment higher for cultural traits that are associated with model biases compared to traits associated with content biases?
Income	Does higher income result in a higher or lower dependency on natural resources, thus reflecting differences in knowledge?	[94, 95]	Context biases lead people to choose information that is more adaptive to their living conditions. Differences in income result in different cultural trait selection biases, which result in population heterogeneity.	Are people with high incomes in a human population models for copying adaptive information? Are the cultural traits that are associated with people with lower incomes copied less often?
Educational level	Can access to higher education affect local ecological knowledge?	[73, 96]	Higher education reflects access to new cultural traits, which can compete with the cultural traits of local ecological knowledge and may be selected due to model or content biases.	Can the promotion of formal education in human populations lead to a reduction in cultural traits that are associated with natural resource use?
Urbanization	Can access to the knowledge and services that originate from urbanization promote a decrease in knowledge regarding natural resources?	[30, 97]	Urbanization introduces variations in cultural traits through migration. Many of these new traits compete with preexisting cultural traits.	Does the information that originates from urbanization occupy the same cultural domain as preexisting traits? How do these distinct cultural traits (which result from urbanization and social-ecological systems) interact over time?
Human perception	How does human perception of the environment affect the resources that will be known or used?	[98, 99]	Different people may have different cultural selection biases and different transmission pathways that depend on their individual or social perception. Therefore, perception affects the mechanisms that are used by people to recognize the more adaptive information.	How does the perception of a given resource or cultural trait affect the copying of information? Are the natural resources that are similarly perceived by different human populations used for the same purposes?
Knowledge transmission	How does the process of knowledge transmission occur? How do different transmission pathways generate changes in knowledge?	[5, 6, 69, 70]	Different transmission pathways result in different speeds of information transmission within a system.	How does knowledge transmission occur in different environments? Which cultural traits tend to be conserved? If these pathways remain predominant in the system, how does knowledge within the system occur over time?

### Cultural selection and cultural drift: competition and chance among cultural traits

In addition to information variation, processes that favor the most beneficial traits within a range of diverse traits are necessary for culture to be adaptive (see [17]). Mathematical models indicate that the transmission of cultural traits must be selective. Otherwise, the added evolutionary benefit of a cultural system compared to an individual learning system would not be guaranteed [16]. In the aforementioned example of resource redundancy, some resources within a range of redundant but useful resources may be preferred over others [7, 40, 42, 44, 59]. From a CE perspective, this preference reflects the competition for expression; a preferred cultural trait is simply a more attractive trait in a given social-ecological system. When an individual indicates his preference for one useful species over another, it is because the information regarding this species is more competitively expressed than the information regarding the other species with the same function (redundant). The competition for expression will result in cultural selection, which results from biases in the memorization and copying of specific cultural traits.

Several types of biases have been described [9, 12, 39], and we can categorize them as content and context biases. Content biases consider the intrinsic attractiveness of a trait, which is usually predetermined by biological or prior cultural evolution [9, 13]. Context biases indicate that the copying of specific cultural traits depends on the learning environment, the learner’s condition/life experiences (i.e., the frequency of the trait in a population), or the success of the individual (model) who possesses the trait (i.e., success bias) [9, 39].

The ways in which content biases influence the dynamics of cultural traits in a population can be characterized by the intrinsic preferences of different groups when copying information, such as the preference for transmitting social information versus non-social information [60], the preference for transmitting information that evokes feelings of disgust [61], or the adaptive memory that forms when information is memorized [62, 63]. Adaptive memory is a form of memory that has evolved to help individuals remember useful and relevant information during decision-making processes, where information that is more advantageous in terms of adaptation is more easily memorized and recalled. The concept of adaptive memory is extremely relevant to EE research [2], and it describes why some traits become fixed in a population rather than others. However, differential fitness can vary between individuals or human groups, and a trait that appears more advantageous for one person or group is not necessarily adaptive in the cultural context for another person or group.

To explain content biases (i.e., the attractiveness of a cultural trait), Mesoudi [9] draws attention to what Rogers [64] calls “attributes of innovations and their rate of adoption.” According to Rogers [64], an innovation will be adopted if it is advantageous over other information, if it is compatible with the preexisting system, and if it is testable and observable to others. In ethnobiological studies, we can observe several characteristics of attractive cultural traits that focus on the drivers of preferences for information regarding useful plants. For example, an investigation of medicinal plant preferences by Ferreira Júnior et al. [42] shows that the preferred plant is the more effective one, which has an advantage over the less effective alternative. Santoro et al. [7] have supplemented these data with their observations that in the absence of the preferred medicinal species, the second- and third-ranked preferences are other medicinal plants, even when other remedy types for treatments exist, such as industrial drugs; this shows that biomedical information can be incompatible with medical systems and is less preferred. In a study of plants used for fuel, Ramos et al. [65] found that people may share more information regarding the plants with higher heating values (i.e., a higher number of transference events) than the plants with lower heating values (i.e., with low efficiencies of heat production).

These studies do not address preferences in the context of cultural evolution, and we can only assume that the preference characteristics indicate content biases. An important question that arises from these results is whether people are aware of their preference choices. For example, in a study of plants used for food, Henrich and Henrich [66] observe that pregnant women avoid dangerous fish, which can cause miscarriages, without explicitly understanding this link. They simply copy the information from other people (i.e., models, who are usually family members or experts) without understanding the reason for specifically avoiding the food. In this case, the copying behavior is not guided by content bias; it is guided by context. The reproductive state of the pregnant woman can be the context that biases the copying of information [39], but it is evident in this study that the context bias is model-based and dependent on the people who possess the information.

Some studies have shown that information transmission depends on models with high expertise [36, 67]. The selection of the model to be copied usually occurs before the content of the information and its advantages and disadvantages are known [9]. In these cases, copying information from people with expertise in a particular field can be adaptive because expertise indicates that the person is a specialist or possesses great knowledge on the subject. For example, in a society that is sustained by hunting, the best way to learn about hunting is by copying the actions of the hunters with the most knowledge because they can direct the best strategies to guarantee successful hunting. However, the information source is often unrelated to the information that is copied. People may perceive a person’s success in a specific field and begin copying cultural traits from this person that are associated with other fields through an associative process [36]. For example, people who accumulate high expertise in a community by being good fishermen may be copied in other domains of knowledge, such as crop cultivation (see [36]).

Model-based context is not always guided by expertise; it can be age-based (preference for copying people of a specific age), gender-based (preference for copying women or men), kin-based (preference for copying family members), and so on (see [39]). In the context of medicinal plant information, ethnobiological studies have shown that people mostly acquire knowledge at home with their parents [5, 6, 68–70], which may indicate the importance of the kin-based model bias in local medical systems. Additionally, the ethnobiological literature is replete with examples that show differences in the local ecological knowledge between people of different ages (where older people have more knowledge than younger people) [71, 72], education level (where a high education level is associated with less ecological knowledge) [73] and gender (where women have more knowledge on specific cultural contexts while men have it on others) [73–78]. These parameters can also be used to verify a group’s tendency to use models from a particular gender, age, or educational level for copying information. Table 4 presents several possible questions that arise from these studies. For example, Pfeiffer and Butz [79] suggest that when knowledge differences exist between genders, the knowledge variations can be strongly influenced by transmission of the knowledge between the genders and by gender-based differences in the social network, which can be explained by the model-based bias of gender dependence.

When there is no clear incentive toward or advantage to copy one trait or one good model over another, people tend to copy information that is shared by the majority of people in a population [13, 80–82]. This tendency is called frequency-dependent or conformity bias [9, 13] and assumes that if a person does not know how to behave, it is more advantageous to behave in a similar manner as the majority. For example, an individual may prefer to copy a more common house structure when building a house in a rural community because he does not know the most effective of all the available possibilities. Here, he can see that a relatively large number of successfully built houses in this rural community indicate that the probability that this particular house structure will fall is low. Conformity bias can be observed when the copy frequency of the majority exceeds what is probabilistically expected by randomly copying the behavior [13]. The opposite of this tendency is anti-conformity, when people prioritize copying the least disseminated cultural trait in a population.

It is important to note that the fixation, extinction, or variation in the frequency of cultural traits can occur both by competition and selection of traits and by chance, in a process known as cultural drift, which is analogous to genetic drift. These traits can be introduced into a population by random variation, guided variation, or migration; they do not undergo any form of selection, but they vary widely in frequency, which results in their fixation or elimination [9]. Like genetic drift, cultural drift is a more important process in smaller populations because chance-sampling accidents are more probable. However, the occurrence of cultural drift in human groups appears unlikely because many cultural trait variation dynamics are guided, and there are several cognitive and psychological biases that influence the choices that are made at the time of transmission; however, some studies have shown that several cultural traits are randomly distributed within populations [83, 84].

### Heredity in cultural evolution: the transmission of cultural traits

Cultural transmission may be the main microevolutionary process of interest in ethnobiology (i.e., [5, 6, 56, 69, 70]). However, these studies present different viewpoints regarding teaching and learning, which specifically result in methodological and analytical differences.

Several studies have characterized learning as a complex process in which individuals become competent at a given cultural skill. For example, Bock [85] observes that the development of competency for a specific task occurs from an energy investment trade-off between the development of body characteristics and the investment in learning experiences. This perspective is similar to the learning model that is proposed by Ruddle and Chesterfield [86], which describes that the learning of a complex skill occurs through a sequence of different tasks, which begin with the simple tasks that are mediated by trial-and-error events. Other studies have analyzed learning as the result of specific events of information transference (see [5, 69]). For example, Soldati et al. [6] have investigated whether learning strategies are influenced by the characteristics of the environment where people live; they address the initial assumption that learning occurs during specific events over the informants’ lifetimes, and they have quantified this information.

Because CE is an approach that originates from mathematical models of population genetics and epidemiology (see [9, 22]), it emphasizes the importance of the frequency of information within a population. Therefore, CE indicates that the specific acquisition of information (cultural traits) should be prioritized over the process of becoming more proficient in a given skill. These broad-level transmission pathways are easier to model than skill proficiency, and CE favors the construction of testable models and hypotheses regarding cultural transmission.

As previously mentioned, parent-to-offspring cultural transmission may not always occur. Mesoudi [9] has subdivided cultural transmission into the following three distinct pathways: (1) from the parents to the offspring (vertical), (2) between unrelated members of the same generation (horizontal), and (3) between unrelated members of different generations (oblique). The scopes of the distinct transmission pathways are related to their possible spread. For example, the horizontal and oblique pathways have a higher scope than the vertical pathway because they allow the transmission of traits between all individuals of the same generation or of different generations [9]. Hewlett and Cavalli-Sforza [27] have subdivided these pathways into the following two types: “one-to-many” and “many-to-one.” In “one-to-many” transmission, traits are transmitted from a model, such as a teacher, leader, or the media (television or radio), to multiple individuals in one group, who are usually students or apprentices. In “many-to-one” transmission, the typically older members of the social group teach one selected individual. However, the scope of vertical transmission is limited to the number of offspring of a given parent [13] and can often be “one-to-one” transmission (Table 3).

Ethnobiological studies have found that the cultural traits of medical systems are mainly transmitted through vertical transmission [5, 6, 68–70]. This form of transmission suggests that plant medicinal resource information varies little over time. Vertical transmission is typically more conserved because transmission from parents to offspring only results in the accumulation of changes from one generation to the next. However, the horizontal and oblique pathways have wider scopes and can promote quick changes, which are often observed in the cultural trait changes that result from knowledge transmission from the media.

The method that is used to collect the data regarding an information transmission pathway should be chosen carefully. For example, people tend to overestimate their learning from parents when simply asked about who taught them about medicinal plants [70, 87–89]. Several studies of vertical transmission have suggested that although the information may have been perfected through horizontal or oblique pathways during the informants’ lifetimes (an essential process in the dynamics of local knowledge), the informants may have only referred to their first contact with the medicines, which often occurred during childhood [36].

In addition to the transmission pathway and scope, an important aspect of the study of cultural transmission within a CE framework is the transmission mechanism. According to Mesoudi [9], cultural traits can be transmitted through a *particulate* mechanism, where a trait is transmitted completely from one person to another, or through *blending*, where one individual can simultaneously adopt different proportions of two competing traits (Table 3). The particulate mechanism resembles what occurs with gene transmission, where information is transmitted by discrete units. One example is the information that plant *X* can be used as fuel for fire pits. People can faithfully transmit this information to one another without interference from another source. The approach of discrete units of cultural information has been widely used in memetics [10], where cultural evolution can be viewed through a neo-Darwinian perspective.

The blending mechanism can be better understood by considering the transformative process through which cultural information is transmitted; the transmission reflects a variation gradient, whereby each trait can be expressed to a varying degree. Transmission by blending is similar to the transmission that occurs for phenotypic variations, in that intermediate phenotypes result from the partial dominance of one gene or the co-dominance of two genes [90]. People usually blend cultural information from various sources before they transmit it to other people. An example of cultural transmission by blending is the belief system of an indigenous group after being colonized and its subsequent acquisition of information regarding Christianity, which resulted in a generation of new religious doctrines through the blending of original indigenous beliefs with Christian dogmas. Nevertheless, we believe that even continuous variation that is caused by blended transmission can be analyzed as discrete units in specific cases. For example, the blending of indigenous and Christian beliefs can be studied as a particulate mechanism; people can adopt a set of binary traits from the Christian belief system and maintain the indigenous beliefs for other traits. This phenomenon is frequently observed with syncretism. Furthermore, for the statistical analysis, it is helpful to divide a continuous variation into discrete categories (i.e., divide the continuous scale into “high,” “medium,” and “low”), but whether the loss of information is worth the advantage of analyzing discrete data remains unclear. Nevertheless, it is necessary to collect large amounts of continuous data to observe this type of blending as a particulate mechanism.

This article is not intended to deepen the polemic between the vision of cultural transmission as a preservative mechanism, where discrete traits are transmitted with high fidelity and the vision that information is reconstructed each time; it is transmitted as a result of blending (for this discussion, see [14]). However, we must clarify that we do not favor the neo-Darwinian perspective in the understanding of cultural evolution; we prefer the Darwinian perspective. Therefore, a cultural trait (discrete or not) is one aspect of the human phenotype and is the basis for the analysis of human evolution (see [18]). However, evolution occurs at the level of the organism and is not based on the replicator, whether it is a gene or a cultural trait. Thus, ethnobiological studies usually focus on *particulate* transmission because it is often difficult, if not impossible, to collect and analyze large amounts of continuous data, such as that for traits that are transmitted by *blending*. A well-studied example is the *particulate* transmission of discrete “plant-therapeutic treatment” traits in the studies of medicinal plants.

## Maladapted cultural traits

Multiple microevolutionary processes may lead to a dissemination of traits that are not necessarily advantageous. The existence of maladapted cultural traits goes against what is expected of human fitness (i.e., these cultural traits do not positively contribute from an adaptive point of view and should, therefore, be eliminated during the selection process) [13]; however, they tend to remain in human populations. For example, in the context of medical systems, Tanaka et al. [31] argue that the treatments that are disseminated in a human population are not always the most effective. These ineffective and unsafe traditional drugs, which are likely to be transmitted, can be considered maladapted cultural traits. Why do maladapted cultural traits arise become established and undergo transmission?

According to CE, maladapted cultural traits arise as a collateral effect of information transmission strategies that allow individuals to obtain lower-cost adaptive information [91]. Thus, maladaptation results from evolutionary trade-offs by offering the possibility for individuals of a human population to cheaply and rapidly acquire adaptive cultural information, while simultaneously allowing the establishment and propagation of variants that fail to increase fitness [13].

Therefore, people may acquire any common behavior as long as it does not clearly conflict with their personal inferences. If there are cognitive or social processes that make maladapted information common (such as conformity bias and cultural drift) and if that information is not overtly false or damaging, people will copy it [19]. Therefore, the studies that focus only on a system’s adaptive characteristics will fail to understand the real nature of human behavior [13]. If all cultural traits are adaptive, why do traits that do not add to the genetic fitness of human populations remain? This question should be the subject of future studies on the use of natural resources by human populations.

## Conclusions

In this article, we discuss the potential of CE to supply a theoretical and explanatory framework to better understand the evolutionary processes that affect social-ecological systems, and this approach may help to predict their behavior over time. Table 4 summarizes several processes and factors that may affect social-ecological systems, as well as the possible contributions of CE to these discussions in EE. This summary highlights future research opportunities toward understanding how social-ecological systems evolve; additionally, this research may contribute to the growth of ethnobiology as a science. Finally, these studies on social-ecological systems may generate new insights into the processes of cultural evolution by serving as empirical evidence of CE.

## Abbreviations

CE: Cultural evolution
EE: Evolutionary ethnobiology
